# The Tudor Domain-Containing Protein BbTdp1 Contributes to Fungal Cell Development, the Cell Cycle, Virulence, and Transcriptional Regulation in the Insect Pathogenic Fungus Beauveria bassiana

**DOI:** 10.1128/spectrum.00564-21

**Published:** 2021-08-11

**Authors:** Lei Qiu, Ze Li, Li Zhang, Tong-Sheng Zhang, Shun-Juan Hu, Ji-Zheng Song, Jia-Hua Liu, Jing Zhang, Juan-Juan Wang, Wen Cheng

**Affiliations:** a State Key Laboratory of Biobased Material and Green Papermaking, Qilu University of Technology, Shandong Academy of Sciences, Jinan, China; b School of Biological Science and Technology, University of Jinan, Jinan, China; c Maize Research Institute, Shandong Academy of Agricultural Sciences, Jinan, China; Broad Institute

**Keywords:** entomopathogenic fungi, SND1, cell cycle, pathogenicity

## Abstract

Beauveria bassiana is an insect pathogenic fungus that serves as a model system for exploring the mechanisms of fungal development and host-pathogen interactions. Clinical and experimental studies have indicated that *SND1* is closely correlated with the progression and invasiveness of common cancers as a potential oncogene, but this gene has rarely been studied in fungi. Here, we characterized the contributions of an SND1 ortholog (Tdp1) by constructing a *BbTdp1* deletion strain and a complemented strain of B. bassiana. Compared with the wild-type (WT) strain, the Δ*BbTdp1* mutant lost conidiation capacity (∼87.7%) and blastospore (∼96.3%) yields, increased sensitivity to chemical stress (4.4 to 54.3%) and heat shock (∼44.2%), and decreased virulence following topical application (∼24.7%) and hemocoel injection (∼40.0%). Flow cytometry readings showed smaller sizes of both conidia and blastospores for Δ*BbTdp1* mutants. Transcriptomic data revealed 4,094 differentially expressed genes (|log_2_ ratio| > 2 and a *q* value of <0.05) between Δ*BbTdp1* mutants and the WT strain, which accounted for 41.6% of the total genes, indicating that extreme fluctuation in the global gene expression pattern had occurred. Moreover, deletion of *BbTdp1* led to an abnormal cell cycle with a longer S phase and shorter G_2_/M and G_0_/G_1_ phases of blastospores, and enzyme-linked immunosorbent assay confirmed that the level of phosphorylated cyclin-dependent kinase 1 (Cdk1) in the Δ*BbTdp1* strain was ∼31.5% lower than in the WT strain. In summary, our study is the first to report that BbTdp1 plays a vital role in regulating conidia and blastospore yields, fungal morphological changes, and pathogenicity in entomopathogenic fungi.

**IMPORTANCE** In this study, we used Beauveria bassiana as a biological model to report the role of BbTdp1 in entomopathogenic fungi. Our findings indicated that BbTdp1 contributed significantly to cell development, the cell cycle, and virulence in B. bassiana. In addition, deletion of *BbTdp1* led to drastic fluctuations in the transcriptional profile. BbTdp1 can be developed as a novel target for B. bassiana development and pathogenicity, which also provides a framework for the study of Tdp1 in other fungi.

## INTRODUCTION

As a widely used insect pathogenic fungus, Beauveria bassiana has become a novel model organism for the analysis of fungal development, pathogenesis, and interactions with hosts (1, 2). Transcription factors (TFs) are considered critical proteins involved in the pathogenicity of fungi (3). B. bassiana TFs are widely distributed and implicated in multiple biological processes (4, 5). For example, the MADS-box protein Mcm1 serves as the central TF in cell cycle regulation, and deletion of *Mcm1* represses the expression of corresponding cellular event-involved genes, which further leads to abnormal cell development, morphology, and pathogenesis as well as a disordered cell cycle (6). BbMbp1, a component of the MluI cell cycle box-binding complex, has been shown to significantly contribute to fungal morphological changes, cell development, and virulence by regulating the transcription of the downstream targets *BbCwp* and *BbImp* (7). Furthermore, the Zn(II)_2_Cys_6_ TF Thm1 specifically functions in mediating heat and cell wall stress by altering membrane structure to participate in virulence (8). As key upstream activators of the central developmental pathway, BrlA and AbaA are considered major controllers of conidia production, dimorphic transition, and insect pathogenicity (9); however, there are still many unreported TFs involved in the regulation of fungal pathogenicity. Research on these TFs will help improve the fungal pathogenic regulatory network and enhance fungal biocontrol potential.

Tudor domain-containing protein (Tdp1; a homologous protein of SND1) has been proven to be an evolutionarily conserved regulator composed of four staphylococcal nuclease (SNc) domains, providing endonuclease activity for SND1 and a tudor domain with the extraordinary ability to interact with nucleic acids and proteins (10). Originally, SND1 as a transcriptional coactivator was relevant for the regulation of gene expression-related processes as well as for the mediation of the dynamic balance of proteins and lipids (10, 11). SND1 has also been shown to participate in cell cycle regulation by mediating the G_1_/S phase transition (12, 13). Over the years, clinical and experimental studies have shown that, as a potential oncogene, *SND1* expression is positively correlated with the progression and invasiveness of many common cancers, suggesting that *SND1* may be a novel candidate gene for tumor therapy and an important indicator of prognosis (11, 14). Moreover, in Drosophila melanogaster, SND1 recognizes and binds to methyl-arginine/lysine residues to significantly participate in various epigenetic interactions, gene expression, and the regulation of various small RNAs (15). In Entamoeba histolytica, SND1 has been confirmed to be a multifunctional protein involved in transcription, stress responses, signaling, metabolism, and human pathogenicity (16, 17).

However, SND1/Tdp1 has rarely been studied in fungi. Hence, we used B. bassiana to illuminate the roles of Tdp1 through polymorphic phenotypic, pathogenicity, and transcriptional analyses of a *BbTdp1* disruption mutant (Δ*BbTdp1*) compared to control strains, including the wild-type (WT) strain and a complemented strain (Δ*BbTdp1*/*BbTdp1*). Our results demonstrated that Tdp1 is required to maintain fungal cell growth, morphological changes, and the cell cycle and also participates in infection processes.

## RESULTS

### Identification of BbTdp1 in B. bassiana and generation of its mutant strains.

*BbTdp1* (locus tag, BBA_02313) encoded a putative 883-amino acid protein containing four SNc domains and one tudor domain in B. bassiana (Fig. S1A in the supplemental material). Phylogenetic analysis revealed that BbTdp1 had higher homology in fungi belonging to entomopathogens and phytopathogens than homology in animal pathogens and yeast fungi (Fig. S1B), but no orthology was observed in Saccharomyces cerevisiae. To explore the roles of BbTdp1 in B. bassiana, the Δ*BbTdp1* and Δ*BbTdp1/BbTdp1* strains were generated as described in Materials and Methods. The results were verified through PCR amplification and quantitative real-time PCR analysis (Fig. S1C and D).

### Effect of BbTdp1 on colony growth and hypha extension.

**FIG 1:**
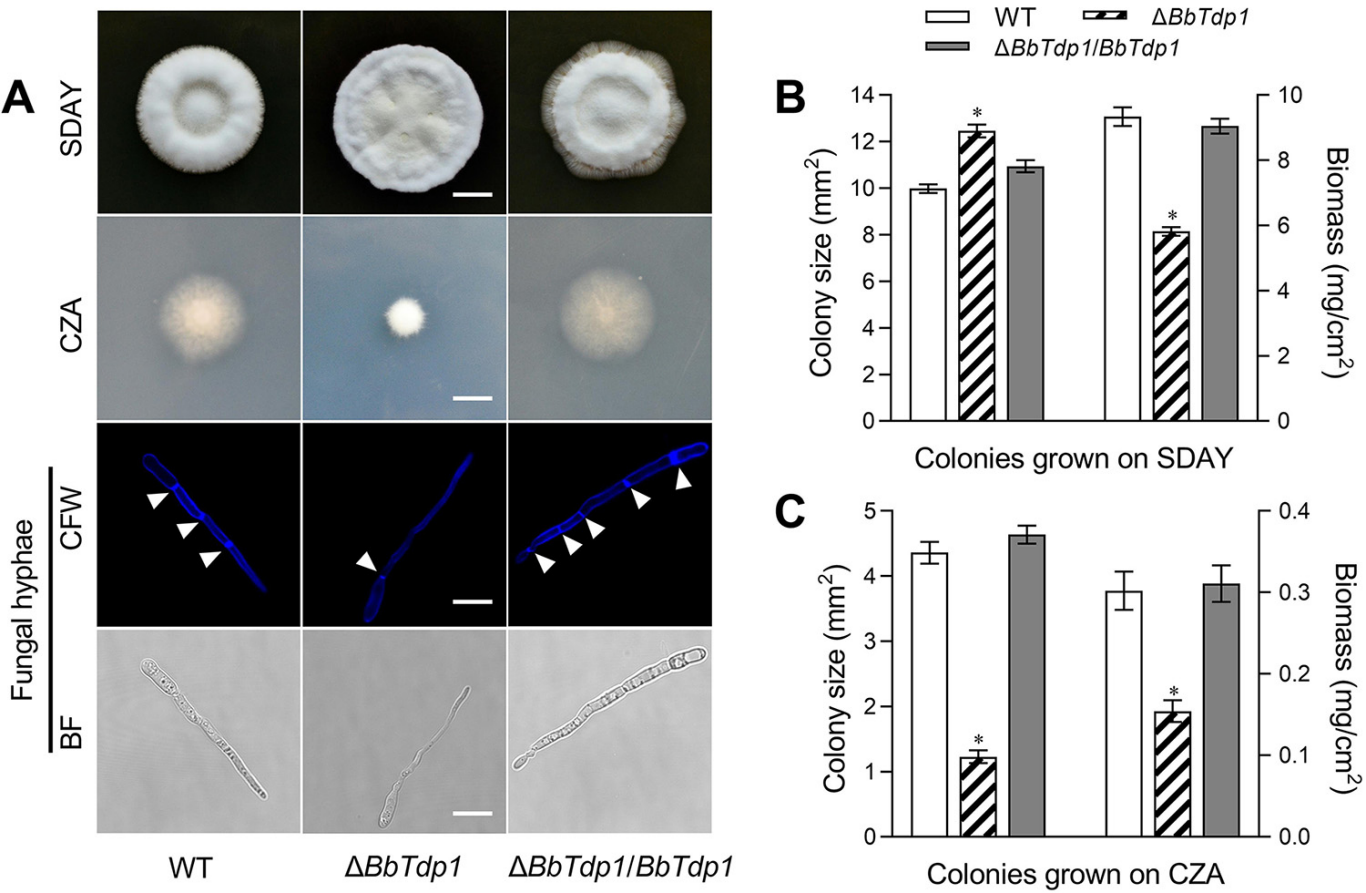
Assays of colony growth and fungal hyphae. (A) Images of colonies grown on SDAY and CZA plates (scale bars, 5 mm) and confocal microscopy images of hyphae (scale bars, 5 μm). Note that the arrows represent the hyphal septation. (B, C) Colony size and hyphal biomass of strains grown on SDAY and CZA plates. Error bars represent standard deviations (SDs) of three replications (*, *P < *0.05).

After culturing for 10 days, colonies on the medium of different nutrient sources showed obvious differences. For the enrichment medium (SDAY), the Δ*BbTdp1* colony was ∼24.7% larger than the WT colony, whereas the hyphal dry weight of the Δ*BbTdp1* colony grown for 10 days was ∼37.7% lighter than that of the WT. For the sterile medium (CZA), the colony areas of the Δ*BbTdp1* strain displayed a decrease of ∼71.8% compared with the WT, and, similarly, the hyphal biomass of the Δ*BbTdp1* strain was ∼49.0% lighter than that of the WT (Fig. 1A to C). In terms of hyphal micromorphology, Δ*BbTdp1*-mutant hyphae were thinner than those of the WT strain and Δ*BbTdp1*/*BbTdp1* strains, and the stained Δ*BbTdp1*-mutant hyphae showed sparser septa than those of the WT under the fluorescence field (Fig. 1A). All the above data indicated abnormal colony growth and hyphae extension in the Δ*BbTdp1* strain.

### Roles of BbTdp1 in conidial development and quality.

**FIG 2:**
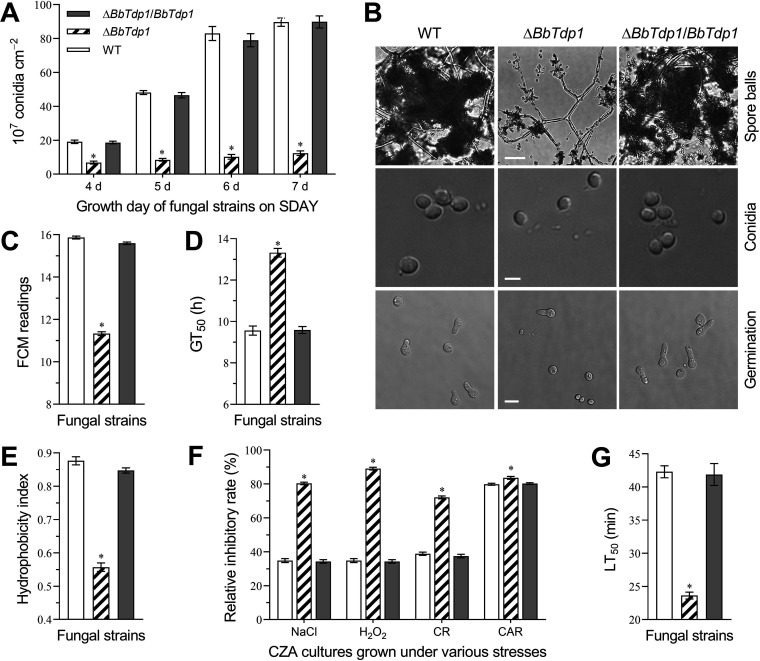
Determination of conidium yield and properties. (A) Conidium yield of fungal strains cultured on normal SDAY plates at 25°C. (B) Confocal microscope images of spore balls (scale bars, 20 μm), conidia (scale bars, 2 μm), and the conidia under germination (scale bars, 4 μm). (C) Conidia size assessed by FCM readings of 1 × 10^4^ conidia per sample. (D) Mean conidial germination time of 50% (GT_50_). (E) Cell surface hydrophobicity index of conidia grown on SDAY medium for 6 days. (F) Chemical stress inhibitory rate of colonies grown on CZA plates. (G) Mean conidial half-lethal time (LT_50_) under 45°C heat stress. Error bars represent SDs of three replications (*, *P < *0.05).

To investigate the effects of *BbTdp1* ablation on conidial development and quality, we performed a multiphenotypic analysis. For the Δ*BbTdp1* strain, conidiation was significantly lower than that of the WT strain from day 4 to day 7, and this gap reached ∼87.7% on day 6 (Fig. 2A), which also could be confirmed by analysis of the spore balls (Fig. 2B). Confocal microscopy showed that the size of the Δ*BbTdp1*-mutant conidia was smaller than that of the WT conidia (Fig. 2B), which was in agreement with the flow cytometry (FCM) readings showing that conidial size decreased by ∼28.6% in the Δ*BbTdp1* strain compared to the WT strain (Fig. 2C).

The half-germination time (GT_50_; an indicator of germination ability) of the Δ*BbTdp1* strain was ∼39.3% more delayed than that of the WT (Fig. 2D), which meant that the Δ*BbTdp1* strain required more time for conidial germination than WT (Fig. 2B). Conidial surface hydrophobicity, a cell surface characteristic, has become a vital factor for conidial infection of fungi against hosts (18). The test results showed that the conidial hydrophobicity of the Δ*BbTdp1* strain (0.56 ± 0.02) was ∼36.4% lower than that of the WT strain (0.88 ± 0.02) (Fig. 2E).

Stress response experiments were performed to measure the cellular sensitivity of the Δ*BbTdp1*-mutant strain and two control strains to osmolarity (NaCl), oxidation (H_2_O_2_), cell wall disturbance (Congo red [CR]), or fungicide (carbendazim [CAR]) chemical stress using the relative inhibition rate. Compared with the control strains, the Δ*BbTdp1* strains showed more sensitivity to chemical stress. For the relative inhibition rate, the measurement results demonstrated that NaCl, H_2_O_2_, CR, and CAR were ∼45.6%, ∼54.3%, ∼33.2%, and ∼4.4% more inhibitive, respectively, to the Δ*BbTdp1* strain on day 10 (Fig. 2F). Conidial thermotolerance was evaluated by the half-lethal time (LT_50_) after heat shock at 45°C. The LT_50_ of the Δ*BbTdp1* strain (23.6 ± 0.7 min) was ∼44.2% lower than that of the WT strain (42.3 ± 1.3 min), which meant that the Δ*BbTdp1* strain was more sensitive to thermal stress (Fig. 2G).

### Contribution of BbTdp1 to blastospore yield and size.

**FIG 3:**
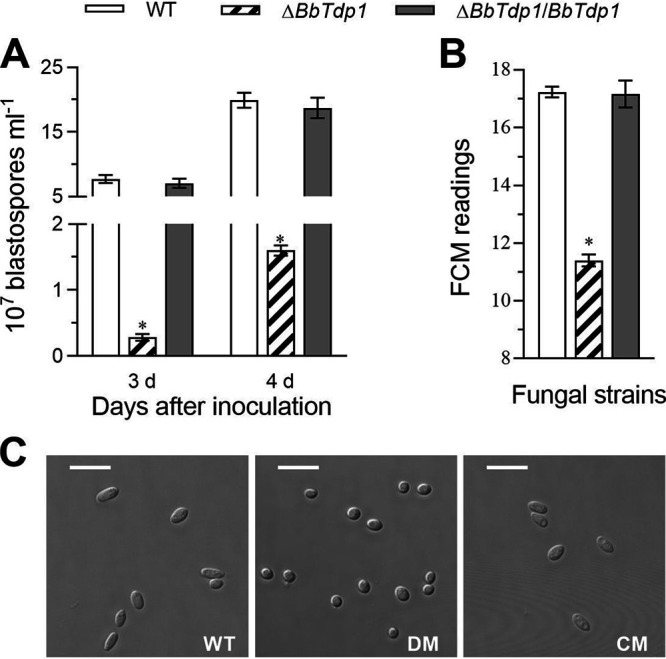
Assessment of blastospore yield and features. (A) Blastospore yield of fungal strains cultured in NLB at 25°C. (B) Blastospore size assessed by FCM readings of 1 × 10^4^ blastospores per sample. (C) Images of blastospores under a confocal microscope (scale bars, 5 μm). Error bars represent SDs of three replications (*, *P < *0.05). DM, disruption mutant; CM, complemented mutant.

After successful infection, B. bassiana survived in insect hemocoels as unicellular blastospores. We used nitrogen-limited broth (NLB) medium to simulate the insect hemocoels for producing blastospores, and the results showed that the blastospore yield of the Δ*BbTdp1* strain was far below the yield observed in the WT strain. When cultivated for 3 days, the blastospore yield of the Δ*BbTdp1* strain was ∼96.3% less than that of the WT strain (Fig. 3A). Furthermore, from the FCM readings, the blastospore size of the Δ*BbTdp1* strain was ∼33.8% smaller than that of the WT strain (Fig. 3B). In addition, confocal microscopy revealed a small blastospore size for Δ*BbTdp1* mutants, and the blastospores also had an ellipsoidal shape, whereas the normal blastospores of the WT strain appeared fusiform (Fig. 3C).

### Requirements of BbTdp1 for virulence.

**FIG 4:**
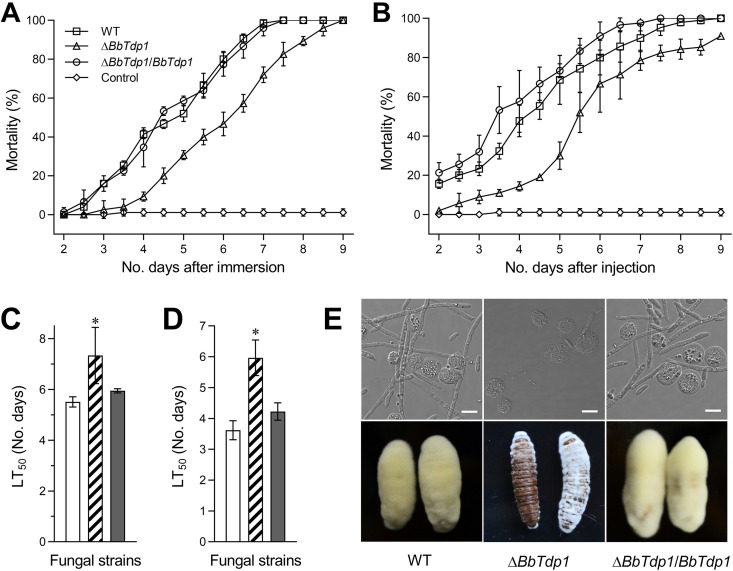
Measurement of fungal infection virulence in G. mellonella larvae. (A, B) Mortality of larvae (from 2 to 9 days) by topical immersion and hemocoel injection, respectively. (C, D) Mean larval half-lethal time (LT_50_) by topical immersion and hemocoel injection, respectively. (E) Confocal microscopy images (top, scale bars represent 5 μm) of mycelia in insect body fluids beginning 3 days after injection (the strip and short rod-like structures are fungal hyphae and blastospores, and the spherical and ellipsoidal shapes are insect hemocytes). Larval surface changes at 5 days after death (bottom). Error bars represent SDs of three replications (*, *P < *0.05).

Topical application and hemocoel injection were used to distinguish the resistance of insect cuticle. Regardless of whether infection occurred *in vivo* or *in vitro*, the larvae infected by the Δ*BbTdp1* mutant died significantly more slowly than larvae infected with the two control strains (Fig. 4A and B). For topical application, the LT_50_ was 7.3 ± 0.9 days for Δ*BbTdp1* mutants, which was ∼24.7% longer than that observed in WT (5.5 ± 0.2 days) (Fig. 4C); moreover, for hemocoel injection, the LT_50_ of Δ*BbTdp1* mutants was 6.0 ± 0.5 days, which was ∼40.0% longer than that of WT (3.6 ± 0.3 days) (Fig. 4D). At 3 days postinjection, the insect hemolymph was removed and observed. Δ*BbTdp1* strains produced a smaller quantity of *in vivo* blastospores and shorter mycelia than the control strains (Fig. 4E). When the insect cadavers were cultured to day 5, the hyphal growth of the larval surface infected by the Δ*BbTdp1* strains was significantly slower than that of the larvae killed by the control strains (Fig. 4E).

### Analysis of the BbTdp1 transcriptional profile.

**FIG 5:**
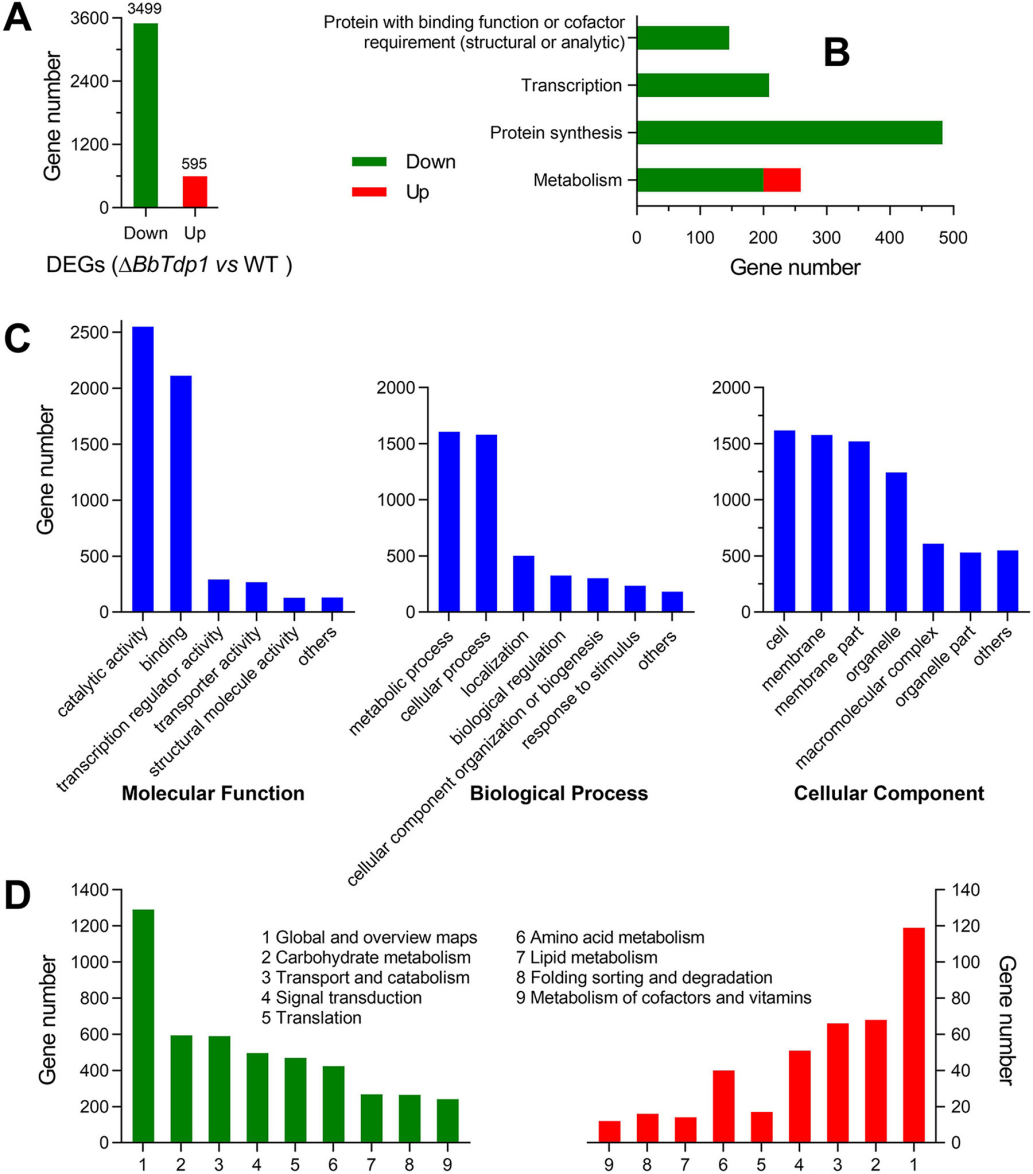
Changes in the BbTdp1-mediated gene transcription profile. (A) Number of downregulated and upregulated DEGs (|log_2_ ratio| > 2, *q* value of <0.05). (B) Functional category (FunCat) classification of DEGs between Δ*BbTdp1* and WT strains. (C) GO classification of DEGs between Δ*BbTdp1* and WT strains, including primary levels of molecular function, biological process, and cellular component. (D) KEGG pathway classification of downregulated DEGs and upregulated DEGs between Δ*BbTdp1* and WT strains.

High-throughput RNA sequencing was used to analyze the differences between the WT strain and Δ*BbTdp1* mutants. All the clean tags, which corresponded to a total of 9,852 genes obtained after filtering, were mapped to the genome of B. bassiana (19); among them, 9,305 genes were shared by both strains. The transcriptomic data indicated that the disruption of *BbTdp1* led to an altered expression pattern of 4,094 differentially expressed genes (DEGs; |log_2_ ratio| > 2 and *q* value of <0.05), with 3,499 downregulated and 595 upregulated genes in the Δ*BbTdp1*-mutant strain compared with the WT strain (Fig. 5A). The DEGs accounted for 41.6% of the total number of genes, which is a very large fluctuation in the transcriptional profile, suggesting that the deletion of *BbTdp1* caused a drastic fluctuation to strain function.

Functional category (FunCat) analysis revealed that the downregulated DEGs (Fig. 5B; Table S2) were enriched in the following categories: (i) protein with binding function or cofactor requirement (structural or analytic), including genes involved in RNA binding; (ii) transcription, including genes related to rRNA synthesis, rRNA processing, tRNA processing, and tRNA modification; (iii) protein synthesis, including massive numbers of genes associated with translation, ribosomal proteins, and ribosome biogenesis (e.g., ribosomal proteins, eukaryotic translation initiation factors, tRNA synthetases, helicases, and WD domain-containing proteins); and (iv) metabolism, including many genes associated with nucleotide degradation and metabolism. The upregulated DEGs in the Δ*BbTdp1*-mutant strain were enriched only in secondary metabolism within the metabolism category; these genes were members of the cytochrome P450 family or encoded major facilitator superfamily transporters and ABC multidrug transporters (Fig. 5B; Table S3).

For Gene Ontology (GO) classification, the total DEGs were divided into three primary categories, including molecular function, biological process, and cellular component (Fig. 5C). At the level of molecular function, most DEGs were mapped into the terms of catalytic activity, binding, transcription regulator activity, and transporter activity, and at the biological process level, there were more DEGs belonging to metabolic process and cellular process (Fig. 5C). Moreover, DEGs associated with cell, membrane, and organelle were classified at the level of cellular component (Fig. 5C). Based on the analysis of the above GO classification, the deletion of *BbTdp1* may lead to abnormities of enzyme catalytic activity, metabolic processes, and cellular components. In terms of KEGG pathway analysis, both downregulated DEGs and upregulated DEGs were almost sorted into nine pathways covering global and overview maps, carbohydrate metabolism, transport and catabolism, signal transduction, translation, amino acid metabolism, lipid metabolism, folding, sorting, and degradation, and metabolism of cofactors and vitamins (Fig. 5D).

From the analysis of the transcriptome, knockout of *BbTdp1* caused the suppression of a large proportion of genes involved in the metabolism of carbohydrates, amino acids, and lipids, catalytic activity, protein synthesis and transport, and signal transduction, which suggested global regulation by BbTdp1 in B. bassiana.

### Ablation of *BbTdp1* alters the cell cycle.

**FIG 6:**
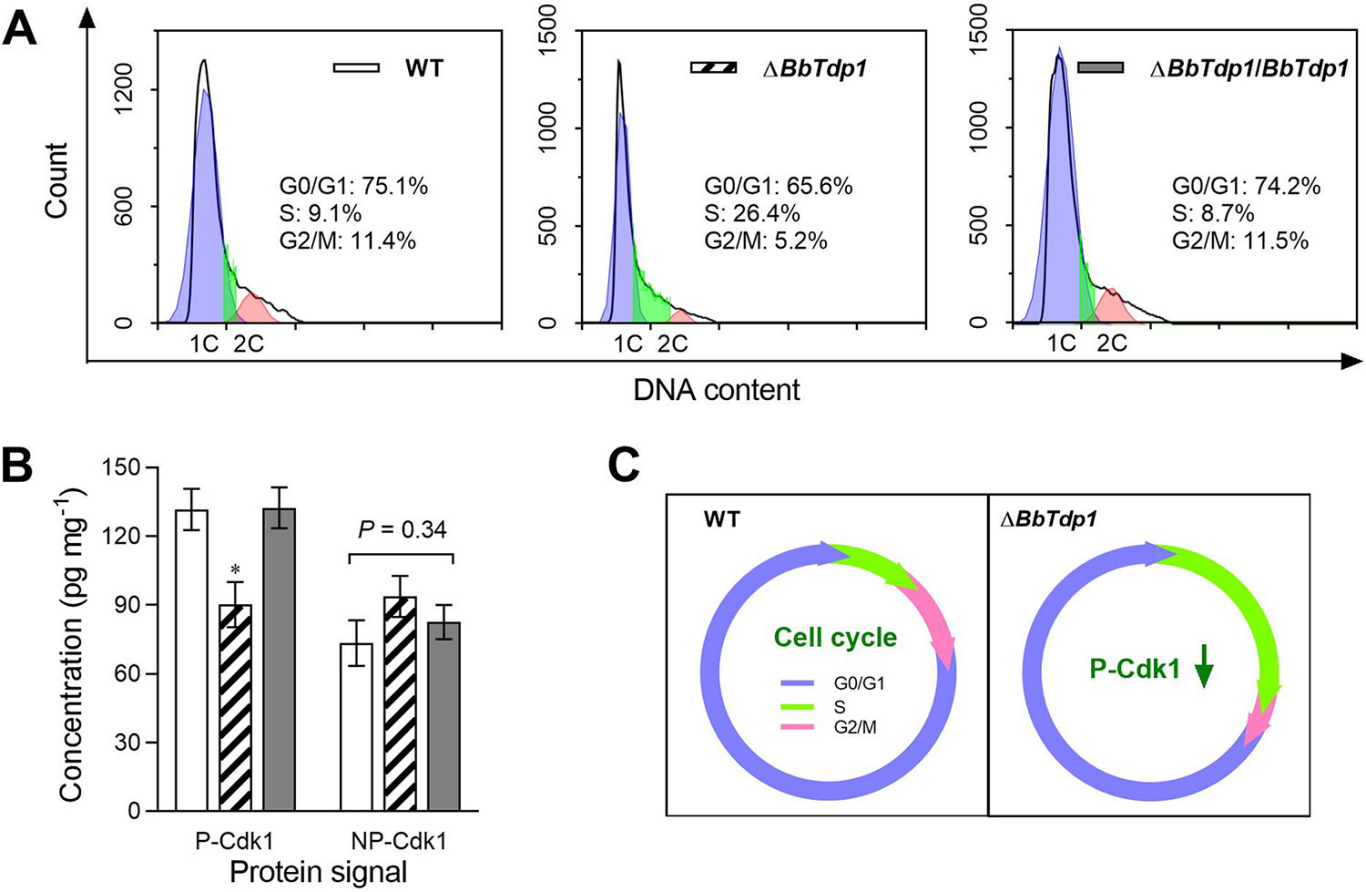
Examination of fungal cell cycle. (A) Cell cycle (G_0_/G_1_, S, and G_2_/M phases) of blastospores indicated by unduplicated (1C), duplicated (2C), and intermediate DNA content profiles according to FACS analysis. (B) The concentration of phosphorylated and nonphosphorylated Cdk1 protein (P-Cdk1 and NP-Cdk1, respectively) in each strain measured by ELISA. Error bars represent SDs of three replications (*, *P < *0.05). (C) The knockout of *BbTdp1* destroyed the balance ratio between phosphorylated and unphosphorylated Cdk1, causing alterations of the cell cycle of Δ*BbTdp1*-mutant fungal cells.

Fluorescence-activated cell sorter (FACS) analysis revealed that in terms of the blastospore cell cycle, Δ*BbTdp1* mutants had a significantly (∼65.6%) longer S phase, an ∼54.4% shorter G_2_/M phase, and an ∼12.6% shorter G_0_/G_1_ phase than the control strains, suggesting that more Δ*BbTdp1*-mutant cells were blocked in the S phase than the other phases and they failed to enter the G_2_ phase (Fig. 6A).

To further verify the role of BbTdp1 in the cell cycle, we used enzyme-linked immunosorbent assay (ELISA) technology to detect the levels of phosphorylated and unphosphorylated cyclin-dependent kinase 1 (Cdk1), which are necessary to maintain the normal cell cycle in B. bassiana (20). The results showed that the concentration of phosphorylated Cdk1 in the Δ*BbTdp1* strain was ∼31.5% lower than in WT, while the concentration of nonphosphorylated Cdk1 showed no significant difference among the three strains (Fig. 6B).

## DISCUSSION

Similar to other SND1 orthologs (e.g., TSN, Tudor-SN, and p100), BbTdp1 contains four SNc domains and one tudor domain. As a transcription coactivator, SND1 is considered to be involved not only in the regulation of gene expression, such as RNA splicing, interference, stability, and editing, but also in the control of protein and lipid homeostasis (10). In human cells, mature functional microRNAs (miRNAs) are degraded by SND1, which promotes the progression of the cell cycle, indicating that targeting SND1 nuclease activity could inhibit cell proliferation (13). Clinically, SND1 is considered a marker of malignant cancer because it is overexpressed in a variety of primary tumors and invasive cancer cells (14, 21). Here, BbTdp1 was characterized to be involved in the mediation of cell development, the cell cycle, and virulence and causes various alterations in the transcription patterns of corresponding cellular event-associated genes in B. bassiana.

In nature, B. bassiana mainly exists in the form of aerial conidia whose yield and quality are extremely important for fungal pathogenicity (1, 22). Lower conidiation in Δ*BbTdp1*-mutant strains than in WT strains may be associated with the downregulation of the transcriptional activity of related genes (Table 1). FluG proteins are required for the synthesis of the extracellular conidiation-inducing factors, and the *FluG*-null mutant showed profuse aerial growth but no conidiation (23). The conidiation process is also controlled by a central developmental pathway composed of the BrlA, AbaA, and WetA (Table 1) regulatory factors in B. bassiana (9). As a C_2_H_2_-zinc finger-type TF, BrlA controls the progression and completion of conidiation (9, 24). AbaA proteins with a TEA/ATTS DNA-binding motif are considered dimorphic transition switches with BrlA proteins (9), and WetA has a conserved ESC/WetA-related domain that contributes to conidiation and secondary metabolism (25). In addition, delayed germination and relatively small cell size were observed for the Δ*BbTdp1* strain, which may be the reason for the decreased virulence. The positive correlation of pathogenicity with spore size and germination speed has been confirmed to be highly significant (26). In Mucor circinelloides, strains with larger spores were more virulent, which may be associated with the germination ability of spores (27). When the pathogenicity of different *Mucorales* species to Galleria mellonella was investigated, a positive correlation was found between spore size and virulence for the same species (28).

**TABLE 1:** Associated genes identified from the BbTdp1-mediated transcriptional profile

Gene category/name	Locus tag	Annotation	log_2_ ratio	Q-value
Conidial development				
*FluG*	BBA_04942	FluG protein	−8.6	9.65e-81
*BrlA*	BBA_07544	zinc finger protein	−4.7	0
*AbaA*	BBA_00300	FoabaA-like protein	−6.2	0
*WetA*	BBA_06126	hypothetical protein	−5.4	0
Blastospore proliferation				
*Cdk1*	BBA_02861	Cyclin-dependent protein kinase	−2.9	0
*Wee1*	BBA_08543	mitosis inhibitor protein kinase	−1.0	2.94e-740
Cell wall integrity				
*Hyd1*	BBA_03015	class I hydrophobin	−5.9	0
*Pmt1*	BBA_07762	mannosyltransferase	−2.9	0
*Pmt2*	BBA_07508	mannosyltransferase	−2.4	0
*Pmt4*	BBA_00053	*O*-mannosyl-transferase	−2.4	0
*Ktr1*	BBA_07003	α-1,2-mannosyltransferase	−1.8	2.07e-192
*Ktr4*	BBA_00842	α-1,2-mannosyltransferase	−3.5	0
*Kre2*	BBA_08768	glycolipid 2-alpha-mannosyltransferase	−3.0	0
Insect epidermal degradation				
*CDEP-1*	BBA_00443	cuticle-degrading protease	−0.9	0
*Chi2*	BBA_02381	endochitinase Chi2	−2.9	0
Superoxide dismutation				
*Sod1*	BBA_02311	Cu/Zn superoxide dismutase	−3.1	0
*Sod2*	BBA_09706	cytosol Mn-superoxide dismutase	−0.8	1.65e-247
*Sod3*	BBA_09382	mitochondrial manganese superoxide dismutase	−1.8	0
*Sod4*	BBA_04317	superoxide dismutase	−1.4	7.52e-141
*Sod5*	BBA_01984	superoxide dismutase	−1.9	0
Secondary metabolism				
*Ops1*	BBA_08179	polyketide synthase	−1.2	6.11e-17
*Bsls*	BBA_02630	bassianolide nonribosomal peptide synthetase	−4.4	2.23e-7

In the insect hemocoel, entomopathogenic fungi are present in the form of unicellular blastospores and fight against the host immune system through cell proliferation (29, 30). When blastospores were cultured under simulated conditions, the yield of blastospores was dramatically reduced in the Δ*BbTdp1* mutant, indicating that *BbTdp1* contributes to blastospore proliferation. In previous studies of oncogenes, *SND1* was found to be highly expressed during the stage of malignant proliferation and aggressiveness of many common human cancer cells (10). In colon cancer, SND1 overexpression significantly accelerated cell proliferation in the exponential growth phase (31). In hepatocellular carcinoma, SND1 has been shown to be involved in controlling cell growth and proliferation (32). Furthermore, compared with the WT strain, the Δ*BbTdp1*-mutant strain displayed a longer S phase and shorter G_2_/M and G_0_/G_1_ phases of blastospores, demonstrating that the disruption of *BbTdp1* induced cell cycle arrest during the S phase and delayed entry into the G_2_/M phase (Fig. 6C). In human tumor cells, Cdk1 promoted the phosphorylation of ribose-phosphate diphosphokinase 1 (PRPS1) at S103, and the loss of phosphorylation at S103 altered the cell cycle, which was similar to the alteration of the cell cycle observed in Δ*BbTdp1* mutants (33). In Schizosaccharomyces pombe, the Ser/Thr protein kinase Cdk1 is an important factor in the cell cycle pathway that controls the G_2_/M phase transition by mediating the phosphorylation of Tyr15 (34, 35). The knockout of *BbTdp1* destroyed the balance ratio between phosphorylated and unphosphorylated Cdk1, causing alterations in the cell cycle of the Δ*BbTdp1* strain (Fig. 6C). In B. bassiana, the balance of Cdk1 dephosphorylation and phosphorylation are coordinated by the phosphatase cell division cycle 25 (Cdc25) and the kinase Wee1 (20). The Δ*Wee1* mutant displays smaller blastospores, a delayed cell cycle, and reduced virulence, confirming that the change in virulence had a linear relationship with blastospore size (20), and these changes were consistent with the changes in blastospores in the Δ*BbTdp1*-mutant strain. Hence, the defects in virulence in the Δ*BbTdp1*-mutant strain were partly attributable to blastospore alterations, such as slow proliferation and aberrant cell cycle and size.

BbTdp1 is indispensable for fungal virulence, which is reflected not only in cell development but also in the cell infection process. First, the conidia of Δ*BbTdp1*-mutant cells had lower hydrophobicity and a more fragile cell wall than those of WT cells, and they were therefore sensitive to cell wall interference agents. Hydrophobin (Table 1) forms a rodlet layer on the conidial surface in B. bassiana, and the inactivation of *Hyd1* causes decreased spore hydrophobicity and altered surface carbohydrate epitopes, reducing their virulence (16). The disruption of *BbTdp1* significantly repressed the expression of *O*-mannosyltransferases (i.e., Pmt1, Pmt2, and Pmt4) and α-1,2-mannosyltransferases (i.e., Ktr1, Ktr4, and Kre2), which have been confirmed to maintain fungal cell wall integrity and pathogenicity (36, 37). Second, the insect body wall is composed of cuticles and chitins; therefore, the downregulation of cuticle-degrading protease (CDEP-1) and chitinase (Chi2) may be the reason for the reduced virulence. CDEP-1 of B. bassiana enhances virulence due to the contribution of conidial germination and cuticle penetration (38). In Isaria fumosorosea, the disruption of *Chi2* led to an apparent delay in insect lethality, suggesting that chitinase is required for fungal penetration of the insect body wall and is a crucial virulence factor (39). Third, Δ*BbTdp1* strains exhibited sensitivity to oxidative stress, which may be attributed to the downregulated transcription of superoxide dismutase (SOD; Table 1), which is involved in scavenging intracellular reactive oxygen species (ROS) *in vivo* (40). In B. bassiana, all five SODs have been reported to be vital for antioxidation and virulence, and among them, BbSod2/3 (i.e., cytosol manganese superoxide dismutase and mitochondrial manganese superoxide dismutase, respectively) dominates the total SOD activity (40, 41). Fourth, BbTdp1 contributed to virulence by mediating the transcription of genes associated with secondary metabolism (e.g., oosporein and bassianolide). Oosporein, a secondary metabolite synthesized by the polyketide synthase cluster (PKS) oosporein synthase, facilitates hyphal infection by inhibiting host immunoreaction responses, and when the *BbPKS9* gene is interrupted, oosporein production is lost and virulence is reduced (42). Another secondary metabolic toxin bassianolide, which is synthesized by the nonribosomal peptide synthetase BbBsls, has been shown to significantly contribute to virulence in B. bassiana (43).

## MATERIALS AND METHODS

### Strains, media, and growth conditions.

All strains of B. bassiana ARSEF 2860 were maintained in sand tubes stored at −80°C and cultured at 25°C in SDAY enrichment medium (4% glucose, 1% peptone, 1% yeast extract, and 1.5% agar). Fungal stress responses were assayed in sterile CZA medium (3% sucrose, 0.3% NaNO_3_, 0.1% K_2_HPO_4_, 0.05% KCl, 0.05% MgSO_4_, and 0.001% FeSO_4_) with different chemical stresses (20).

### Analysis of structure and phylogenetics.

The *BbTdp1* gene (NCBI accession code EJP68311.1) from the genome of B. bassiana was explored via BLAST within the NCBI database (http://blast.ncbi.nlm.nih.gov/Blast.cgi) (19). The identified BbTdp1 protein was structurally compared with other queried representative entomopathogens, phytopathogens, animal pathogens, and yeast by means of the simple modular architecture research tool (SMART) database at http://smart.embl-heidelberg.de. Phylogenetic analysis was completed by using the neighbor-joining method in MEGA 7 software (http://www.megasoftware.net) (44).

### Generation of BbTdp1 disruption and complemented strains.

Disruption and complemented strains were constructed according to our previously described method (45–47). Recombination events were verified via PCR and quantitative real-time PCR, and primers used to build the knockout and complemented strains are included in Table S1 in the supplemental material.

### Measurement of colonies and hyphae.

For observations of nutritional requirements, colonies of Δ*BbTdp1* mutants and two control strains were started by spotting 1 μl of a 10^7^ conidia ml^−1^ suspension (the same concentration below unless specified) on the centers of SDAY and CZA plates (48), and, meanwhile, aliquots (100 μl) of conidia suspensions were spread on SDAY and CZA plates with cellophane overlaid evenly for the determination of biomass (hyphal dry weight).

To assess hyphal septation and morphology, hyphae were harvested from SDB (agar-free SDAY) cultures at 25°C for 48 h and stained with calcofluor white (Sigma) at ambient temperatures for 15 min. A laser scanning confocal microscope (Leica DMi8, Germany) was used to observe the stained hyphae under the bright/fluorescent fields (wavelength, excitation = 335 nm and emission = 433 ± 5 nm) (6).

### Assessment of conidial development and quality.

Aliquots (200 μl) of a 10^7^ conidia ml^−1^ suspension were spread on SDAY plates with cellophane overlaid evenly. Three culture slices (5-mm diameter) were drilled daily from each plate using a borer and were crushed for suspension in 1 ml of 0.02% Tween 80; the plugs were counted using a hemocytometer at 1-day intervals from day 4, and the counts were calculated as the number of conidia per cm^2^ to assess the conidiation capacity (45).

Conidia harvested from 6-day-old SDAY plates were suspended in phosphate-buffered saline (PBS) solution (pH 7.4) for cell size analysis measured by FCM readings (49) using FACS analysis of 1 × 10^4^ cells per sample in an FC500 MCL flow cytometer (Beckman Coulter, CytoFLEX LX, USA).

Aliquots (100 μl) of conidial suspensions were inoculated into 900 μl of germination broth (GB; 2% sucrose, 0.05% peptone, and 0.02% Tween 80). The half-germination time (GT_50_) was used to measure the conidial germination capacity (45). The microbial adhesion to hydrocarbons method (50) was used to assess the index of the surface hydrophobicity for conidia cultured on SDAY plates after 6 days.

Moreover, a laser scanning confocal microscope was used to provide intuitive visualizations of the bright field for conidial development. Moderate conidial suspension, conidiiferous structures (spore balls) from 6-day SDAY plates, and the conidial suspension after 12 h of germination were observed under a confocal microscope.

One-microliter aliquots of conidial suspensions were pointed to the CZA plates with gradients of NaCl (30 mg ml^−1^), H_2_O_2_ (2 mM), CR (30 μg ml^−1^), and CAR (0.2 μg ml^−1^) as chemical stressors. Colony diameters were determined on day 10, and the relative inhibitory rate was calculated using the formula (Sc − St)/Sc × 100% (i.e., Sc is the area of the colonies grown on CZA plates, and St is the area of the colonies grown on CZA plates with treatments) (51). Conidial thermotolerance was examined by exposing conidial samples to 45°C heat stress followed by a modeling analysis of relative mortality rates using LT_50_ estimates of each strain (52).

### Examination of blastospores and the cell cycle.

To examine blastospore production, 100-μl aliquots of conidial suspensions were inoculated into 50 ml of nitrogen-limited broth (NLB; 4% glucose, 0.4% NH_4_NO_3_, 0.3% KH_2_PO_4_, and 0.3% MgSO_4_) and incubated at 25°C and 160 rpm for 4 days. During this period, blastospore yield was calculated under the microscope every 24 h from day 3. After 4 days, blastospores obtained from NLB were suspended in PBS solution (pH 7.4), and blastospores were observed using confocal microscopy and the size was further determined using FCM. The cell cycles were analyzed by FACS for DNA concentration readings according to our previously described method (20). The G_0_/G_1_, S, and G_2_/M cell cycle phases were determined based on unduplicated (1C), duplicated (2C), and intermediate DNA concentrations (20).

### Enzyme-linked immunosorbent assay.

Protein samples were extracted from the NLB cultures of fungal strains shaken at 25°C for 4 days and were suspended in 1-ml aliquots of PBS (pH 7.4) supplemented with 10 μl of protease inhibitors and phosphatase inhibitors (Mei5 Biotech, Beijing, China) on an ice bath for 10 min. After centrifuging the mixture for 10 min at 4°C and 10,000 × *g*, the supernatant was then centrifuged for another 10 min and used for ELISA. Levels of phosphorylated and nonphosphorylated Cdk1 (cyclin-dependent kinase 1) were detected with P-Cdk1 and NP-Cdk1 kits (Meimian Biotech, Jiangsu, China). The absorbance values of the treated ELISA plates were detected using a microplate reader (Thermo Fisher Scientific Inc.).

### Fungal virulence bioassays.

Fungal virulence tests were performed with third-instar G. mellonella larvae. Three groups of 30 larvae were soaked in 30 ml of conidial suspensions for 10 s for *in vitro* infection or injected with 1 μl of 5 × 10^5^ conidia ml^−1^ suspensions for *in vivo* infection, after which they were immediately transferred into breathable boxes for normal growth. An equal dose of 0.02% Tween 80 was used as the control for the two treatments. All boxes were incubated at 25°C on a 12-h/12-h photoperiod; in addition, deaths were recorded every 12 h (44). As an indicator for assessing larval mortality, the LT_50_ is a death trend index obtained by probabilistic analysis. When the insects were infected for 3 days, the morphology of fungi in the host was observed under a confocal microscope. The dead larvae carcasses were collected and cultured in a saturated humidity chamber to observe the conditions of larvae after death.

### Sequencing of the BbTdp1-mediated transcriptome (RNA sequencing).

To explore the role of BbTdp1, we performed comparative transcriptional analysis of the WT and Δ*BbTdp1* strains to further understand the effects of BbTdp1 on the overall transcriptional pattern. The WT and Δ*BbTdp1* strains were cultured for 6 days on SDAY plates; subsequently, total RNA was extracted. After purifying the RNA samples, six libraries (i.e., three libraries for the WT strain and three for the Δ*BbTdp1*-mutant strain) were constructed and sequenced on the BGISEQ-500 platform at BGI (Shenzhen, China).

After filtering the raw reads, the clean reads were queried against the B. bassiana genomic database (19). Cufflinks was used to quantify the identified genes based on the expected number of fragments per kilobase of exon per million mapped fragments (53). Then, the DEGs between WT and Δ*BbTdp1* strains were filtered with the Cuffdiff method (54). Significant DEGs met the following conditions: *q* value of <0.05 and a |log_2_ ratio| > 2.

To functionally classify the DEGs, we used FungiFun2 (https://elbe.hki-jena.de/fungifun/) to determine gene functional enrichment (55). The functional categories were significantly enriched when the adjusted *P* value was <0.05 (56, 57). In addition, Gene Ontology and signal pathway classification were performed for describing the function of DEGs more intuitively with the help of GO (http://geneontology.org/) (58) and KEGG (https://www.kegg.jp/) (59).

### Statistical analysis.

All experiments comparing the Δ*BbTdp1* strain and the two control strains, including phenotypic observations, measurements, and virulence, were performed in triplicate and were subjected to one-factor (strain) analysis of variance. In addition, Tukey’s honestly significant difference (HSD) test was applied to differentiate the means of each phenotype.

### Data availability.

The sequence data have been deposited in the NCBI Gene Expression Omnibus (accession no. GSE179456).

## References

[1] Mascarin GM, Jaronski ST. 2016. The production and uses of *Beauveria bassiana* as a microbial insecticide. World J Microbiol Biotechnol 32:177. doi:10.1007/s11274-016-2131-3. 27628337

[2] Mascarin GM, Lopes RB, Delalibera I, Jr., Fernandes EKK, Luz C, Faria M. 2019. Current status and perspectives of fungal entomopathogens used for microbial control of arthropod pests in Brazil. J Invertebr Pathol 165:46–53. doi:10.1016/j.jip.2018.01.001. 29339191

[3] Bultman KM, Kowalski CH, Cramer RA. 2017. *Aspergillus fumigatus* virulence through the lens of transcription factors. Med Mycol 55:24–38. doi:10.1093/mmy/myw120. 27816905 PMC6388973

[4] He ZJ, Song YL, Deng J, Zhao X, Qin X, Luo ZB, Zhang YJ. 2020. Participation of a MADS-box transcription factor, Mb1, in regulation of the biocontrol potential in an insect fungal pathogen. J Invertebr Pathol 170:107335. doi:10.1016/j.jip.2020.107335. 32007504

[5] Wang ZL, Pan HB, Huang J, Yu XP. 2020. The zinc finger transcription factors Bbctf1α and Bbctf1β regulate the expression of genes involved in lipid degradation and contribute to stress tolerance and virulence in a fungal insect pathogen. Pest Manag Sci 76:2589–2600. doi:10.1002/ps.5797. 32077581

[6] Zhao X, Yang XJ, Lu ZY, Wang HF, He ZJ, Zhou GY, Luo ZB, Zhang YJ. 2019. MADS-box transcription factor Mcm1 controls cell cycle, fungal development, cell integrity and virulence in the filamentous insect pathogenic fungus *Beauveria bassiana*. Environ Microbiol 21:3392–3416. doi:10.1111/1462-2920.14629. 30972885

[7] Ding JL, Lin HY, Feng MG, Ying SH. 2020. Mbp1, a component of the MluI cell cycle box-binding complex, contributes to morphological transition and virulence in the filamentous entomopathogenic fungus *Beauveria bassiana*. Environ Microbiol 22:584–597. doi:10.1111/1462-2920.14868. 31743555

[8] Huang SS, Keyhani NO, Zhao X, Zhang YJ. 2019. The Thm1 Zn(II)_2_Cys_6_ transcription factor contributes to heat, membrane integrity and virulence in the insect pathogenic fungus *Beauveria bassiana*. Environ Microbiol 21:3153–3171. doi:10.1111/1462-2920.14718. 31211497

[9] Zhang AX, Mouhoumed AZ, Tong SM, Ying SH, Feng MG. 2019. BrlA and AbaA govern virulence-required dimorphic switch, conidiation, and pathogenicity in a fungal insect pathogen. mSystems 4:e00140-19. doi:10.1128/mSystems.00140-19. 31289140 PMC6616149

[10] Ochoa B, Chico Y, Martinez MJ. 2018. Insights into *SND1* oncogene promoter regulation. Front Oncol 8:606. doi:10.3389/fonc.2018.00606. 30619748 PMC6297716

[11] Gutierrez-Beltran E, Denisenko TV, Zhivotovsky B, Bozhkov PV. 2016. Tudor staphylococcal nuclease: biochemistry and functions. Cell Death Differ 23:1739–1748. doi:10.1038/cdd.2016.93. 27612014 PMC5071578

[12] Su C, Zhang CY, Tecle A, Fu X, He JY, Song J, Zhang W, Sun XM, Ren YY, Silvennoinen O, Yao Z, Yang X, Wei MX, Yang J. 2015. Tudor staphylococcal nuclease (Tudor-SN), a novel regulator facilitating G_1_/S phase transition, acting as a co-activator of E2F-1 in cell cycle regulation. J Biol Chem 290:7208–7220. doi:10.1074/jbc.M114.625046. 25627688 PMC4358140

[13] Elbarbary RA, Miyoshi K, Myers JR, Du PC, Ashton JM, Tian B, Maquat LE. 2017. Tudor-SN-mediated endonucleolytic decay of human cell microRNAs promotes G_1_/S phase transition. Science 356:859–862. doi:10.1126/science.aai9372. 28546213 PMC5551500

[14] Jariwala N, Rajasekaran D, Srivastava J, Gredler R, Akiel MA, Robertson CL, Emdad L, Fisher PB, Sarkar D. 2015. Role of the staphylococcal nuclease and tudor domain containing 1 in oncogenesis (review). Int J Oncol 46:465–473. doi:10.3892/ijo.2014.2766. 25405367 PMC4277250

[15] Ying MY, Chen DH. 2012. Tudor domain-containing proteins of *Drosophila melanogaster*. Dev Growth Differ 54:32–43. doi:10.1111/j.1440-169x.2011.01308.x. 23741747

[16] Calixto-Galvez M, Romero-Diaz M, Garcia-Munoz A, Salas-Casas A, Pais-Morales J, Galvan IJ, Orozco E, Rodriguez MA. 2011. Identification of a polypeptide containing Tudor and staphylococcal nuclease-like domains as the sequence-specific binding protein to the upstream regulatory element 1 of *Entamoeba histolytica*. Int J Parasitol 41:775–782. doi:10.1016/j.ijpara.2011.02.002. 21447339

[17] Cazares-Apatiga J, Medina-Gomez C, Chavez-Munguia B, Calixto-Galvez M, Orozco E, Vazquez-Calzada C, Martinez-Higuera A, Rodriguez MA. 2017. The Tudor staphylococcal nuclease protein of *Entamoeba histolytica* participates in transcription regulation and stress response. Front Cell Infect Microbiol 7:52. doi:10.3389/fcimb.2017.00052. 28293543 PMC5328994

[18] Zhang S, Xia YX, Kim B, Keyhani NO. 2011. Two hydrophobins are involved in fungal spore coat rodlet layer assembly and each play distinct roles in surface interactions, development and pathogenesis in the entomopathogenic fungus, *Beauveria bassiana*. Mol Microbiol 80:811–826. doi:10.1111/j.1365-2958.2011.07613.x. 21375591

[19] Xiao GH, Ying SH, Zheng P, Wang ZL, Zhang SW, Xie XQ, Shang YF, St Leger RJ, Zhao GP, Wang CS, Feng MG. 2012. Genomic perspectives on the evolution of fungal entomopathogenicity in *Beauveria bassiana*. Sci Rep 2:483. doi:10.1038/srep00483. 22761991 PMC3387728

[20] Qiu L, Wang JJ, Ying SH, Feng MG. 2015. Wee1 and Cdc25 control morphogenesis, virulence and multistress tolerance of *Beauveria bassiana* by balancing cell cycle-required cyclin-dependent kinase 1 activity. Environ Microbiol 17:1119–1133. doi:10.1111/1462-2920.12530. 24910927

[21] Navarro-Imaz H, Ochoa B, Garcia-Arcos I, Martinez MJ, Chico Y, Fresnedo O, Rueda Y. 2020. Molecular and cellular insights into the role of SND1 in lipid metabolism. Biochim Biophys Acta Mol Cell Biol Lipids 1865:158589. doi:10.1016/j.bbalip.2019.158589. 31978555

[22] Gao PP, Li MC, Jin K, Xia YX. 2019. The homeobox gene *MaH1* governs microcycle conidiation for increased conidial yield by mediating transcription of conidiation pattern shift-related genes in *Metarhizium acridum*. Appl Microbiol Biotechnol 103:2251–2262. doi:10.1007/s00253-018-9558-4. 30631896

[23] Park HS, Yu JH. 2012. Genetic control of asexual sporulation in filamentous fungi. Curr Opin Microbiol 15:669–677. doi:10.1016/j.mib.2012.09.006. 23092920

[24] Li F, Shi HQ, Ying SH, Feng MG. 2015. WetA and VosA are distinct regulators of conidiation capacity, conidial quality, and biological control potential of a fungal insect pathogen. Appl Microbiol Biotechnol 99:10069–10081. doi:10.1007/s00253-015-6823-7. 26243054

[25] Wu MY, Mead ME, Lee MK, Loss EMO, Kim SC, Rokas A, Yu JH. 2018. Systematic dissection of the evolutionarily conserved WetA developmental regulator across a genus of filamentous fungi. mBio 9:e01130-18. doi:10.1128/mBio.01130-18. 30131357 PMC6106085

[26] Altre JA, Vandenberg JD, Cantone FA. 1999. Pathogenicity of *Paecilomyces fumosoroseus* isolates to diamondback moth, *Plutella xylostella*: correlation with spore size, germination speed, and attachment to cuticle. J Invertebr Pathol 73:332–338. doi:10.1006/jipa.1999.4844. 10222189

[27] Li CH, Cervantes M, Springer DJ, Boekhout T, Ruiz-Vazquez RM, Torres-Martinez SR, Heitman J, Lee SC. 2011. Sporangiospore size dimorphism is linked to virulence of *Mucor circinelloides*. PLoS Pathog 7:e1002086. doi:10.1371/journal.ppat.1002086. 21698218 PMC3116813

[28] Maurer E, Hortnagl C, Lackner M, Grassle D, Naschberger V, Moser P, Segal E, Semis M, Lass-Florl C, Binder U. 2019. *Galleria mellonella* as a model system to study virulence potential of mucormycetes and evaluation of antifungal treatment. Med Mycol 57:351–362. doi:10.1093/mmy/myy042. 29924357 PMC6398984

[29] Lu HL, St Leger RJ. 2016. Insect immunity to entomopathogenic fungi. Adv Genet 94:251–285. doi:10.1016/bs.adgen.2015.11.002. 27131327

[30] Lewis MW, Robalino IV, Keyhani NO. 2009. Uptake of the fluorescent probe FM4-64 by hyphae and haemolymph-derived *in vivo* hyphal bodies of the entomopathogenic fungus *Beauveria bassiana*. Microbiology (Reading) 155:3110–3120. doi:10.1099/mic.0.029165-0. 19542008

[31] Tsuchiya N, Ochiai M, Nakashima K, Ubagai T, Sugimura T, Nakagama H. 2007. SND1, a component of RNA-induced silencing complex, is up-regulated in human colon cancers and implicated in early stage colon carcinogenesis. Cancer Res 67:9568–9576. doi:10.1158/0008-5472.CAN-06-2707. 17909068

[32] Yoo BK, Santhekadur PK, Gredler R, Chen D, Emdad L, Bhutia S, Pannell L, Fisher PB, Sarkar D. 2011. Increased RNA-induced silencing complex (RISC) activity contributes to hepatocellular carcinoma. Hepatology 53:1538–1548. doi:10.1002/hep.24216. 21520169 PMC3081619

[33] Jing XQ, Wang XJ, Zhang T, Zhu WC, Fang Y, Wu HX, Liu XY, Ma D, Ji XP, Jiang YM, Liu K, Chen XZ, Shi Y, Zhang YQ, Shi MM, Qiu WH, Zhao R. 2019. Cell-cycle-dependent phosphorylation of PRPS1 fuels nucleotide synthesis and promotes tumorigenesis. Cancer Res 79:4650–4664. doi:10.1158/0008-5472.CAN-18-2486. 31253668

[34] Gould KL, Nurse PJN. 1989. Tyrosine phosphorylation of the fission yeast *cdc2*^+^ protein kinase regulates entry into mitosis. Nature 342:39–45. doi:10.1038/342039a0. 2682257

[35] Li Z, Pinch BJ, Olson CM, Donovan KA, Nowak RP, Mills CE, Scott DA, Doctor ZM, Eleuteri NA, Chung M, Sorger PK, Fischer ES, Gray NS. 2020. Development and characterization of a Wee1 kinase degrader. Cell Chem Biol 27:57–65. doi:10.1016/j.chembiol.2019.10.013. 31735695 PMC6980656

[36] Wang JJ, Qiu L, Cai Q, Ying SH, Feng MG. 2014. Three α-1,2-mannosyltransferases contribute differentially to conidiation, cell wall integrity, multistress tolerance and virulence of *Beauveria bassiana*. Fungal Genet Biol 70:1–10. doi:10.1016/j.fgb.2014.06.010. 24981201

[37] Wang JJ, Qiu L, Chu ZJ, Ying SH, Feng MG. 2014. The connection of protein *O*-mannosyltransferase family to the biocontrol potential of *Beauveria bassiana*, a fungal entomopathogen. Glycobiology 24:638–648. doi:10.1093/glycob/cwu028. 24727441

[38] Zhang YJ, Feng MG, Fan YH, Luo ZB, Yang XY, Wu D, Pei Y. 2008. A cuticle-degrading protease (CDEP-1) of *Beauveria bassiana* enhances virulence. Biocontrol Sci Technol 18:551–563. doi:10.1080/09583150802082239.

[39] Huang Z, Hao YF, Gao TN, Huang Y, Ren SX, Keyhani NO. 2016. The *Ifchit1* chitinase gene acts as a critical virulence factor in the insect pathogenic fungus *Isaria fumosorosea*. Appl Microbiol Biotechnol 100:5491–5503. doi:10.1007/s00253-016-7308-z. 26910039

[40] Xie XQ, Li F, Ying SH, Feng MG. 2012. Additive contributions of two manganese-cored superoxide dismutases (MnSODs) to antioxidation, UV tolerance and virulence of *Beauveria bassiana*. PLoS One 7:e30298. doi:10.1371/journal.pone.0030298. 22279579 PMC3261187

[41] Li F, Shi HQ, Ying SH, Feng MG. 2015. Distinct contributions of one Fe- and two Cu/Zn-cofactored superoxide dismutases to antioxidation, UV tolerance and virulence of *Beauveria bassiana*. Fungal Genet Biol 81:160–171. doi:10.1016/j.fgb.2014.09.006. 25263710

[42] Feng P, Shang YF, Cen K, Wang CS. 2015. Fungal biosynthesis of the bibenzoquinone oosporein to evade insect immunity. Proc Natl Acad Sci U S A 112:11365–11370. doi:10.1073/pnas.1503200112. 26305932 PMC4568701

[43] Xu Y, Orozco R, Kithsiri Wijeratne EM, Espinosa-Artiles P, Leslie Gunatilaka AA, Patricia Stock S, Molnar I. 2009. Biosynthesis of the cyclooligomer depsipeptide bassianolide, an insecticidal virulence factor of *Beauveria bassiana*. Fungal Genet Biol 46:353–364. doi:10.1016/j.fgb.2009.03.001. 19285149

[44] Kumar S, Stecher G, Tamura K. 2016. MEGA7: molecular evolutionary genetics analysis version 7.0 for bigger datasets. Mol Biol Evol 33:1870–1874. doi:10.1093/molbev/msw054. 27004904 PMC8210823

[45] Qiu L, Wei XY, Wang SJ, Wang JJ. 2020. Characterization of trehalose-6-phosphate phosphatase in trehalose biosynthesis, asexual development, stress resistance and virulence of an insect mycopathogen. Pestic Biochem Physiol 163:185–192. doi:10.1016/j.pestbp.2019.11.016. 31973856

[46] Shao W, Cai Q, Tong SM, Ying SH, Feng MG. 2020. Nuclear Ssr4 is required for the *in vitro* and *in vivo* asexual cycles and global gene activity of *Beauveria bassiana*. mSystems 5:e00677-19. doi:10.1128/mSystems.00677-19. PMC7174636 32317391

[47] Wang DY, Ren K, Tong SM, Ying SH, Feng MG. 2020. Pleiotropic effects of Ubi4, a polyubiquitin precursor required for ubiquitin accumulation, conidiation and pathogenicity of a fungal insect pathogen. Environ Microbiol 22:2564–2580. doi:10.1111/1462-2920.14940. 32056334

[48] Shao W, Cai Q, Tong SM, Ying SH, Feng MG. 2019. Rei1-like protein regulates nutritional metabolism and transport required for the asexual cycle *in vitro* and *in vivo* of a fungal insect pathogen. Environ Microbiol 21:2772–2786. doi:10.1111/1462-2920.14616. 30932324

[49] Tzur A, Moore JK, Jorgensen P, Shapiro HM, Kirschner MW. 2011. Optimizing optical flow cytometry for cell volume-based sorting and analysis. PLoS One 6:e16053. doi:10.1371/journal.pone.0016053. 21283800 PMC3024321

[50] Holder DJ, Kirkland BH, Lewis MW, Keyhani NO. 2007. Surface characteristics of the entomopathogenic fungus *Beauveria* (*Cordyceps*) *bassiana*. Microbiology (Reading) 153:3448–3457. doi:10.1099/mic.0.2007/008524-0. 17906143

[51] Cai Q, Wang JJ, Shao W, Ying SH, Feng MG. 2018. Rtt109-dependent histone H3 K56 acetylation and gene activity are essential for the biological control potential of *Beauveria bassiana*. Pest Manag Sci 74:2626–2635. doi:10.1002/ps.5054. 29704296

[52] Wang JJ, Qiu L, Cai Q, Ying SH, Feng MG. 2015. Transcriptional control of fungal cell cycle and cellular events by Fkh2, a forkhead transcription factor in an insect pathogen. Sci Rep 5:10108. doi:10.1038/srep10108. 25955538 PMC4424799

[53] Trapnell C, Williams BA, Pertea G, Mortazavi A, Kwan G, van Baren MJ, Salzberg SL, Wold BJ, Pachter L. 2010. Transcript assembly and quantification by RNA-seq reveals unannotated transcripts and isoform switching during cell differentiation. Nat Biotechnol 28:511–515. doi:10.1038/nbt.1621. 20436464 PMC3146043

[54] Trapnell C, Hendrickson DG, Sauvageau M, Goff L, Rinn JL, Pachter L. 2013. Differential analysis of gene regulation at transcript resolution with RNA-seq. Nat Biotechnol 31:46–53. doi:10.1038/nbt.2450. 23222703 PMC3869392

[55] Priebe S, Kreisel C, Horn F, Guthke R, Linde J. 2015. FungiFun2: a comprehensive online resource for systematic analysis of gene lists from fungal species. Bioinformatics 31:445–446. doi:10.1093/bioinformatics/btu627. 25294921 PMC4308660

[56] Peng YJ, Ding JL, Feng MG, Ying SH. 2019. Glc8, a regulator of protein phosphatase type 1, mediates oxidation tolerance, asexual development and virulence in *Beauveria bassiana*, a filamentous entomopathogenic fungus. Curr Genet 65:283–291. doi:10.1007/s00294-018-0876-y. 30116891

[57] Qiu L, Zhang J, Song JZ, Hu SJ, Zhang TS, Li Z, Wang JJ, Cheng W. 2021. Involvement of BbTpc1, an important Zn(II)_2_Cys_6_ transcriptional regulator, in chitin biosynthesis, fungal development and virulence of an insect mycopathogen. Int J Biol Macromol 166:1162–1172. doi:10.1016/j.ijbiomac.2020.10.271. 33159944

[58] Ye J, Fang L, Zheng HK, Zhang Y, Chen J, Zhang ZJ, Wang J, Li ST, Li RQ, Bolund L, Wang J. 2006. WEGO: a web tool for plotting GO annotations. Nucleic Acids Res 34:W293–W297. doi:10.1093/nar/gkl031. 16845012 PMC1538768

[59] Kanehisa M, Goto S, Furumichi M, Tanabe M, Hirakawa M. 2010. KEGG for representation and analysis of molecular networks involving diseases and drugs. Nucleic Acids Res 38:D355–D360. doi:10.1093/nar/gkp896. 19880382 PMC2808910

