# Supplementary material for: The Tudor Domain-Containing Protein BbTdp1 Contributes to Fungal Cell Development, the Cell Cycle, Virulence, and Transcriptional Regulation in the Insect Pathogenic Fungus Beauveria bassiana
Source: Microbiol Spectr. 2021 Aug 11;9(1):10.1128/spectrum.00564-21. doi: 10.1128/spectrum.00564-21 (PMC8552692; doi:10.1128/spectrum.00564-21)
Supplement: SUPPLEMENTAL FILE 1 — Supplemental material. Download SPECTRUM00564-21_Supp_1_seq9.pdf, PDF file, 0.3 MB [file spectrum00564-21_supp_1_seq9.pdf]

Supplemental Material FOR Publication

The tudor domain-containing protein BbTdp1 contributes to fungal cell development, the cell cycle, virulence and transcriptional regulation in the insect pathogenic fungus *Beauveria bassiana*

Lei Qiu[1*], Ze Li[1], Li Zhang[1], Tong-Sheng Zhang[1], Shun-Juan Hu[1], Ji-Zheng Song[1], Jia-Hua Liu[1], Jing Zhang[1], Juan-Juan Wang[2*], Wen Cheng[3]

[1] *State Key Laboratory of Biobased Material and Green Papermaking, Qilu University of Technology, Shandong Academy of Sciences, Jinan, China*

[2] *School of Biological Science and Technology, University of Jinan, Jinan, China*

[3] *Maize Research Institute, Shandong Academy of Agricultural Sciences, Jinan, China*

[*]Corresponding author

Juan-Juan Wang: *School of Biological Science and Technology, University of Jinan, Jinan, Shandong 250022, PR China. E-mail: wjj880414@163.com*

Lei Qiu: *State Key Laboratory of Biobased Material and Green Papermaking, Qilu University of Technology, Shandong Academy of Sciences, Jinan, Shandong 250353, PR China. E-mail: qiulei.2005@163.com*

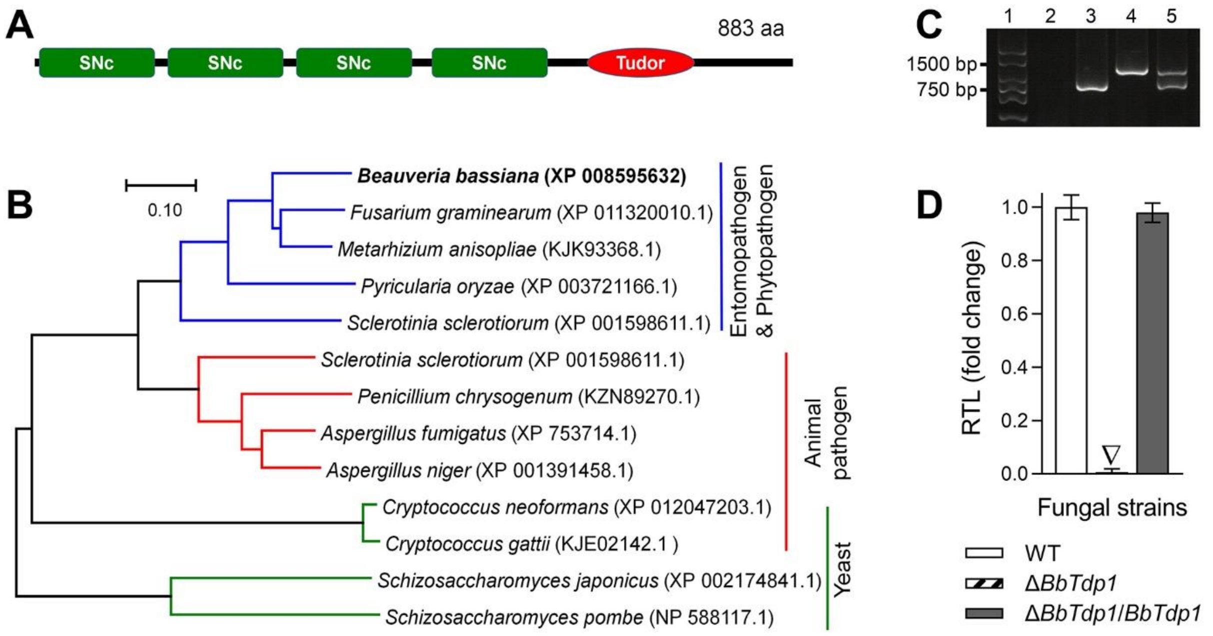

FIG S1 Schematic diagram of the BbTdp1 protein structure and phylogenetic tree and identification of *BbTdp1* mutants. (A) *BbTdp1* encodes a protein of 883 aa, including four staphylococcal nuclease-like domains in tandem followed by a whole tudor domain. (B) Phylogenetic tree of BbTdp1 in *B. bassiana* with its closest fungal orthologues in entomopathogens and phytopathogens, animal pathogens and yeast-like fungi. (C) Detection of *BbTdp1* in WT (lane 3), Δ*BbTdp1* (lane 4), and Δ*BbTdp1/BbTdp1* (lane 5) via PCR in conjunction with primers pairs (Table S1). (D) Relative transcript levels (RTL) of *BbTdp1* in Δ*BbTdp1* and Δ*BbTdp1/BbTdp1* versus the WT standard. Note that the arrow represented that RTL of *BbTdp1* was undetectable in the deletion mutant.

TABLE S1 Primer pairs used for procedures involving *BbTdp1* in *B. bassiana*.

Primers	Paired sequences (5’-3’)*	Purpose
Tdp1up-F/R	aaaGAATTCGCCACCTTGCCTTACCAC/aaaGGATCCGCTTCTCACCGCCGTTTT	Cloning the BbTdp1 5’-end
Tdp1dn-F/R	aaaTCTAGAGCCAAAGAGGAAAAGAAGGG/aaaAGATCTCAGACGGCGACGCTAAAT	Cloning the BbTdp1 3’-end
Tdp1fl-F/R	GGGGACAAGTTTGTACAAAAAAGCAGGCTATGGCGACTACGATGGC/<br>GGGGACCACTTTGTACAAGAAAGCTGGGTTTCGGATTGATTCCTCTGG	Cloning full-length BbTdp1
pTdp1-F/R	aaaTGCTTGGCGAAAAGATGG/aaaTTGCGGCTTGCCTGAC	PCR detecting BbTdp1
qTdp1-F/R	CCGCAGATACACAAGAGA/GCCGTTACCATCAATGTT	qRT-PCR detecting BbTdp1
q18S-F/R	TGGTTTCTAGGACCGCCGTAA/CCTTGGCAAATGCTTTCGC	qRT-PCR detecting 18sRNA

* The underlined regions denote the restriction enzyme sites for *BbTdp1* deletion (*Eco*RI/*Bam*HI and *Xba*I/*Bgl*II) or the fragments of gateway exchange for targeted *BbTdp1* complementation.

TABLE S2 FunCat analysis of the down- regulated DEGs.

TABLE S3 FunCat analysis of the up-regulated DEGs.
